# An integrated approach to the interpretation of Single Amino Acid Polymorphisms within the framework of CATH and Gene3D

**DOI:** 10.1186/1471-2105-10-S8-S5

**Published:** 2009-08-27

**Authors:** Jose MG Izarzugaza, Anja Baresic, Lisa EM McMillan, Corin Yeats, Andrew B Clegg, Christine A Orengo, Andrew CR Martin, Alfonso Valencia

**Affiliations:** 1 Institute of Structural and Molecular Biology, Darwin Building, University College London, Gower Street, London WC1E 6BT, UK; 2 Structural Biology and Biocomputing Programme, Spanish National Cancer Research Centre (CNIO), C/Melchor Fernandez Almagro 3, E28029 Madrid, Spain

## Abstract

**Background:**

The phenotypic effects of sequence variations in protein-coding regions come about primarily via their effects on the resulting structures, for example by disrupting active sites or affecting structural stability. In order better to understand the mechanisms behind known mutant phenotypes, and predict the effects of novel variations, biologists need tools to gauge the impacts of DNA mutations in terms of their structural manifestation. Although many mutations occur within domains whose structure has been solved, many more occur within genes whose protein products have not been structurally characterized.

**Results:**

Here we present 3DSim (3D Structural Implication of Mutations), a database and web application facilitating the localization and visualization of single amino acid polymorphisms (SAAPs) mapped to protein structures even where the structure of the protein of interest is unknown. The server displays information on 6514 point mutations, 4865 of them known to be associated with disease. These polymorphisms are drawn from SAAPdb, which aggregates data from various sources including dbSNP and several pathogenic mutation databases. While the SAAPdb interface displays mutations on known structures, 3DSim projects mutations onto known sequence domains in Gene3D. This resource contains sequences annotated with domains predicted to belong to structural families in the CATH database. Mappings between domain sequences in Gene3D and known structures in CATH are obtained using a MUSCLE alignment. 1210 three-dimensional structures corresponding to CATH structural domains are currently included in 3DSim; these domains are distributed across 396 CATH superfamilies, and provide a comprehensive overview of the distribution of mutations in structural space.

**Conclusion:**

The server is publicly available at . In addition, the database containing the mapping between SAAPdb, Gene3D and CATH is available on request and most of the functionality is available through programmatic web service access.

## Background

The most common biologically-relevant mutations are single base changes often referred to as **single nucleotide polymorphisms **(SNPs). These account for about 90% of sequence polymorphisms in humans [1] at an overall frequency of about one per 1000 bases [2]. Traditionally, SNPs are classified as coding or non-coding according to their genomic location – coding SNPs are further sub-classified according to the protein product expressed. Non-Synonymous SNPs (nsSNPs) are those that alter the amino acid sequence of the protein product, either through amino acid substitution (a 'single amino acid polymorphisms', SAAP), or by the generation of truncation mutations. By contrast, synonymous SNPs (also referred to as silent or sSNPs) are those that do not alter the amino acid sequence of the protein product.

Not all synonymous SNPs are neutral since they may still affect the expression of gene products or protein translation by introducing alterations into regulatory regions, interfering with splice sites or impinging on other regulatory mechanisms [3,4]. Similarly, not all nsSNPs are associated with pathological diseases, since some changes are, by nature, milder than others, and diseases commonly involve complex sets of alterations.

Strictly the term 'SNP' is defined as a mutation which occurs in at least 1% of a 'normal' population. Thus SNPs are expected to have a neutral non-deleterious or low-penetrance phenotypic effect whereas the term **pathogenic deviation **(PD) refers to those mutations that generally occur at much lower frequencies in the population and have a severe effect on phenotype.

The most commonly used database for storing information on SNPs is dbSNP [5], which currently contains several million validated SNPs from humans and other species. Other sources of genomic information about SNPs include Ensembl [6] and the HapMap Project [7].

Several efforts have been devoted to the prediction of the pathogenicity of amino acid mutations, resulting from single nucleotide changes. These methods make use of a set of characteristics which may be based both on sequence and structure, to determine whether a mutation can affect protein function and therefore be, potentially, associated with disease. This is an area of active research as shown by the considerable number of publications on the subject during the last few years [8-18].

Several efforts, SAAPdb [19] among others, have been devoted to compiling this information and to providing a sequence and structural analysis, where possible, aiming to determine the origin of the pathogenicity shown. In this type of repository, the term SNP is used to refer to essentially phenotypically silent mutations, while PD is used for mutations known to have a severe effect on phenotype, i.e. any single base change reported to correlate with disease. Online Mendelian Inheritance in Man (OMIM) [20] is a collection of information about inherited disease and contains data on PDs. However a great deal more information is held and maintained by individual research groups in locus-specific mutation databases or LSMDBs [21]. Like PDs, nsSNPs are point mutations, but by definition they occur in at least 1% of a 'normal' population. They are expected to have a neutral non-deleterious or low-pentrance phenotypic effect whereas PDs are known to be detrimental. By mapping these SAAPs (a term we use for both PDs and mutations resulting from nsSNPs) onto protein structures, we can begin to understand how protein structure might be affected by mutant residues, and so begin to explain the functional effect (if any) of the mutation. SAAPdb provides potential explanations for both PDs, derived from various sources, and SNPs, derived from dbSNP [5].

The CATH [22] structural domain database is a manually curated classification of domain structures found in the Protein Data Bank (PDB) [23], grouped according to evolutionary relationships and structural features. Hidden Markov Models (HMMs) are derived from alignments of these structural exemplars and used by Gene3D [24] to identify homologues within the protein sequences of UniProt [25], RefSeq [20] and Ensembl [6].

Here we present 3DSim (3D Structural Implication of Mutations), a system mapping single amino-acid polymorphisms onto structures of CATH domains. For sequences with no known structure, the Gene3D resource of domain structure annotations is used to map the sequence onto the closest homologous domain of known structure in CATH. Thus 3DSim is of particular interest when no structural information is available for a protein in which mutations are known to occur as it uses sequence homology to map to the closest representative structure. This provides a comprehensive overview of the distribution of mutations in structural space, as well as a visualization tool for pinpointing the locations of mutations on individual structures rendered in Jmol , as well as links to detailed information on each sequence, structure and mutation. The 3DSim application, which was designed with the aim of being very intuitive, easy to use and user-friendly, is publicly available at . Several worked examples are available, along with a 6-minute video tutorial. In addition, for those advanced users needing intensive programmatic access to the information stored, the underlying database containing the mappings between SAAPdb, Gene3D and CATH is available on request, and most of the functionality is available as web services implemented in SOAP.

## Results and discussion

### The mapping between SAAPdb and Gene3D

SAAPdb contains polymorphism data for 11956 sequences without a structure. Almost all of these could be mapped to Gene3D: 11904 identical sequences were found in the Gene3D database. Of the remaining 52, 17 had sequences with the same length and associated uniprot accession, leaving only 35 for which a reliable match could not be obtained directly.

### The mapping between Gene3D and CATH

**Figure 1:**
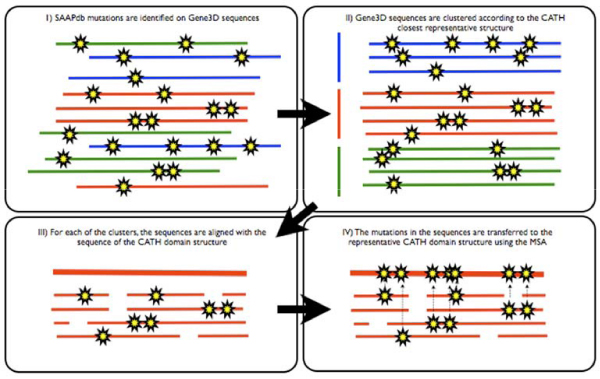
**The mapping pipeline**. Illustration of the mapping process between SAAPdb and CATH via Gene3D

Where no structural data are available, the best representative CATH domain is selected on the basis of homology. For each of the 2179 superfamilies in CATH, a database of all CATH domains was built. For each of the 11904 Gene3D domain sequences mapped to CATH structural superfamilies for which there is information about mutations in SAAPdb (see previous section), a BLAST [26] search was run against the corresponding superfamily database. The closest relative found (i.e. the one with the lowest e-value and highest sequence identity) was used to cluster the sequences. Sequences with a sequence identity less than 20% were placed in separate clusters. This process yielded 2091 different groups. The groups (including the sequence of the representative structure) were then aligned using MUSCLE [27] and the resulting alignments used to transfer the mutations from Gene3D sequences to CATH domain representative structures. At the end of the pipeline we were able to display information on 6514 point mutations, 4865 of them known to be associated with disease, mapping to 396 CATH superfamilies. The complete pipeline is described in Figure 1 and details are provided in the Methods section.

### Description of web application

The initial input for the system is a CATH superfamily identifier for which the user wants to retrieve information on mapped mutations. Alternatively, the database can be searched using Uniprot accession codes or CATH domain identifiers. The user can either manually introduce the desired superfamily identifier in the provided form, or browse the superfamilies in CATH in order to access the information. After this initial step, information about the selected CATH superfamily is displayed along with the CATH domains for which there is information about mutations in SAAPdb.

In addition, for users that are interested in a general overview of the distribution of mutations within structural superfamilies in CATH, one can obtain a list of the superfamilies with known mutations and analyze domains in that superfamily.

Once the user has selected a CATH domain, 3DSim displays both an interactive Jmol plug-in that allows the visualization of the mutations projected onto the three-dimensional structure of the representative CATH domain and a table displaying all the information available for that given domain in terms of available mutations, sequence and structure positions of the mutations, pathogenicity information, and similarity (BLAST sequence identity) between the sequences in Gene3D and the representative CATH domain sequence.

This similarity index provides the user with a hint about the reliability of the homology based transference of mutations between sequences in Gene3D and the structures in CATH. As a rule of thumb, the higher the similarity the more reliable the transference of mutations is. Tweaking this index is of particular interest when there are few mutations in the close relatives for a given structural family and looser constrains need to be taken into account to allow more mutations in the analysis. By default, the server rejects those mutations transferred from sequences obtaining a BLAST sequence identity of less than 20%, but – due to the interactive approach of the server – the user can decide to establish more stringent constrains depending on the study case.

In addition, the site is linked to several external annotation providers (including CATH, Gene3D, SAAPdb, Modbase, PDBsum and UniProt) where more information about the mutations, the proteins and the structures can be gathered. In particular, SAAPdb provides information about the structural implications of mutations. This information can be related, in some cases, to the pathogenic character of the mutations and provides an insight into the mechanism of molecular function for several proteins.

**Figure 2:**
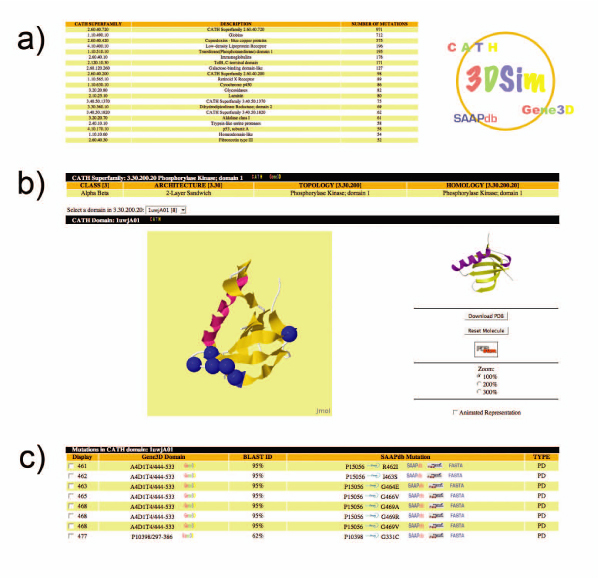
**The web interface**. A collage showing a worked example of the different sections available through the server's graphical user interface. a) Browsable list of superfamilies b) Example of structure displaying known pathogenic mutations c) Explanatory table of the mutations

Figure 2 shows a worked example of the different views available through the server's graphical user interface.

### Description of web services

In order to allow remote programmatic access to the information contained in the database, we have developed a total of nine SOAP web services, powered by the Perl SOAP::Lite toolkit . These allow users to retrieve in simple XML format:

• all known mutations for a given CATH domain, grouped by UniProt ID.

• the total number of mutations in a CATH domain.

• all the CATH domains which are associated with a given UniProt ID.

• the amino-acid sequence of a given CATH domain.

• all CATH domains in a CATH superfamily, queried by the four-part CATH code.

• the superfamily to which a given CATH domain belongs.

• the description and representative structure associated with a given CATH superfamily.

• all the mutations in SAAPdb for a given UniProt accession.

• the total number of mutations in SAAPdb for a given UniProt accession.

These services were designed in such a way as to facilitate construction of computational analysis pipelines. For example, a user starting with a UniProt protein of interest could retrieve a list of all the domains found in that protein, then the CATH superfamilies to which each domain belongs, then all the other domains found in each superfamily, and filly all known mutations in those related domains, by chaining together four web service calls.

More information is provided at . The page contains example Perl code for querying the web-services, and examples of output from each one.

### Database update

The database storing the information presented by both the webserver and the webservice intrinsically depends on the other databases providing the source information (i.e. CATH, Gene3D and SAAPdb), each one being updated at its own pace. This fact, in addition to the computationally expensive calculations needed to compute the mapping between Gene3D and the representative structures in CATH, makes it impossible to schedule an automatic updating calendar. Therefore, the database will be updated based on a release system, where new versions will be made public as regularly as possible.

### Typical usage example

**Figure 3:**
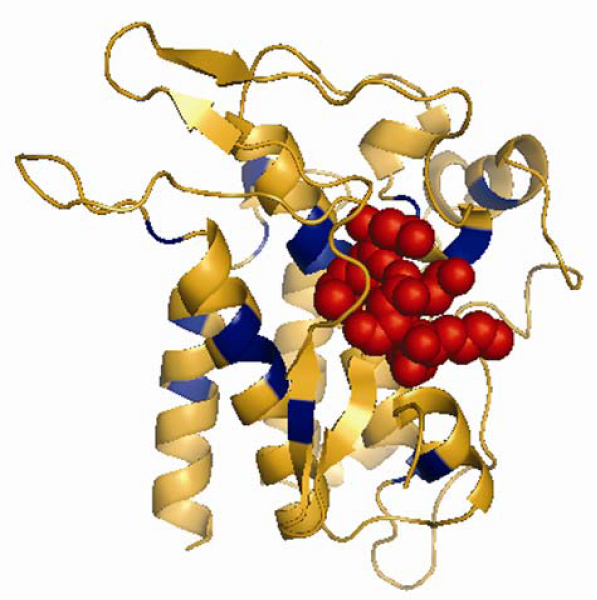
**Three-dimensional structure of CATH domain **1rw8A02. 1rw8A02 is member of the Phosphotransferase superfamily in CATH (1.10.510.10). 24 positions are reported to be pathogenic mutations (PDs, blue).

As an illustrative example, here we present the case of the ATP binding subunit of the kinases (CATH superfamily 1.10.510.10) which is accessible through the server's web page . This superfamily corresponds to the Phosphotransferase domain I homology group in CATH, and is subdivided into a number of different domains. However, for this particular example, we will focus only on the domain with the highest number of mutations (24), 1rw8A02. Of these 24 mutations, only three come from the sequence which maps directly to the domain. The remaining 21 come from homologous sequences with 40–65% sequence identity identified via Gene3D (Table 1). Figure 3 shows the structure with the pathogenic deviations coloured in blue. This image can be obtained directly from the server, and is one of the main features available for the analysis of the distribution of mutations within structures. Additional links to other structure-based databases such as PDBsum [28] are provided in order to enhance the information provided, for this particular case, the position of the catalytic site, involved in binding of ATP, is described to be near residues from 333 to 338. Visual inspection of the position of the pathogenic deviations reveals that they tend to cluster around this catalytic core of the structure. Indeed, the higher the similarity in terms of BLAST identity between the CATH domain and the Gene3D sequence, the closer these positions are to the binding core and hence, more reliable the observations are.

**Table 1:** Mutations mapped to 1rw8A02. SwissProt accession P36897 maps directly to PDB code 1rw8 chain A and to CATH domain 1rw8A02 which represents residues 285–500. Other SwissProt entries containing known pathogenic deviations (PDs) are mapped to this domain via Gene3D and the mutations are mapped to the 1rw8 structure.

SwissProt Accession	Domain Range	Sequence Identity	Mutations
P36897	285–500	100%	M318R, D400G, R487P
O00238	284–499	65%	R486W
P36894	314–529	62%	A338D, C376Y, M470T
P37023	282–497	60%	C344Y, R374W, M376R
			I398N, W399S, R411P
			R411Q, R411W, R484W
P37173	330–546	40%	Y336N, A355P, G357W
			S449F, E526Q, R528C
			R528H, R537C

This PDB chain (1rw8A) maps to the UniProtKB/SwissProt accession P36897 and the information provided by the UniProt record (accessible through the web server's cross references) shows that it corresponds to the TGF-beta receptor type-1 precursor in humans (TGFR1_HUMAN) for which there is a level of association with disease, in particular to Furlong syndrome also known as Loeys-Dietz syndrome type 1A (LDS1); [29]. LDS1 is an aortic aneurysm syndrome with widespread systemic involvement. The disorder is characterized by arterial tortuosity and aneurysms, craniosynostosis, hypertelorism, and bifid uvula (cleft palate). Other findings include exotropy, micrognathia and retrognathia, structural brain abnormalities, intellectual deficit, congenital heart disease, translucent skin, joint hyperlaxity and aneurysm with dissection throughout the arterial tree. The mutations listed as pathogenic deviations (R487P, M318R and D400G) in the server for this protein, which has a 100% identity between the Gene3D sequence and the representative structure of the CATH domain are reported in the literature [29] as involved in LDS1 development.

## Conclusion

We have presented 3DSim (3D Structural Implication of Mutations), a system that enables the localization and visualization of single amino acid polymorphisms projected onto protein structures based on homologous relationships captured in the CATH and Gene3D databases. This provides a comprehensive overview of the distribution of mutations in structural space.

Although there are other servers mapping mutations to structure already (reviewed by Uzun *et al. *[30]) the server presented here has several unique features not available in existing servers. Firstly, the similar treatment of SNPs and the rarer more harmful PDs allows users to inspect and compare both kinds of mutation through the same interface, including explanatory metadata where available. Secondly, the localization of these SAAPs within the CATH hierarchy allows users to query and explore the distribution of mutations at various levels of structural classification. Thirdly, the mapping of sequences onto homologous CATH domains via Gene3D helps users predict the effects of polymorphisms in proteins whose structure has not been solved. Finally, the availability of the data via web services and database dumps enables power users to include this information efficiently in their own analyses. These facilities allow the independent integration of our data in any other pipeline or workflow.

The server has been running internally since we started working on the analysis of point mutations in protein families [31,32] and is accessible at . Examples and documentation are also available, together with a tutorial video and samples of outputs of the main functions. This website is available to all users with no login requirement. It is likely that we will include additional features related with the structural interpretation of mutations and their relationship with disease, after receiving feedback from external users.

In summary the 3DSim server provides up-to-date, complete information automatically to map mutations in the domain sequences of proteins annotated in Gene3D onto protein structures classified in the CATH database.

## Methods

### Obtaining the mutations from SAAPdb

SAAPdb [19] is a database of single amino acid polymorphisms (SAAPs) from several resources, such as dbSNP [5], ADABase [33], G6PD [34] HAMSTeRs [35], IARC p53 Database [36], LDLR [37], OMIM , OTC [38], SOD1db [39] and ZAP70Base [33], mapped to protein structure, where available in the PDB [23]. As of October 2008, SAAPdb contains 9060 unique pathogenic deviations (PDs: SAAPs associated to a disease) and 2532 unique single nucleotide polymorphisms (SNPs: SAAPs with no known pathogenic effect) successfully mapped to the UniProtKB [25] sequences in Gene3D [24].

Both pathogenic deviations and single nucleotide polymorphisms were only taken into account if the alteration introduced was non-silent, that is, if the mutation is both in a coding residue and the resulting amino acid is different from the native one. Where mutations are recorded both as neutral and disease-associated, the mutations were considered pathogenic.

### Gene3D domain assignments

The process by which homologues of CATH domains are identified in sequences, and presented in the Gene3D database, has been described previously [40]. For this particular dataset, the CATH v3.2.0 Hmmer HMM library was scanned against the UniProt (Swiss-Prot and TrEMBL) sequence database in collaboration with the SIMAP database [41]. FASTA files of each superfamily were generated by extracting the subsequences of the domains belonging to each superfamily.

### Gathering the sequences from Gene3D

For each CATH superfamily a library of one or more HMMs is generated using the SAM Target2K procedure [22]. These HMMs are then searched against UniProt in collaboration with the SIMAP resource at the Munich Information Centre for Protein Sequences [41]. The hits are resolved into a single set of non-overlapping domains for each sequence, using the in-house DomainFinder 2.0 protocol. The resulting domain subsequences were then extracted and dumped into the relevant superfamily FASTA file.

### Gathering the sequences of the CATH domains

For each of the 2097 superfamiles in CATH [22], all CATH domains were recovered along with the corresponding amino acid sequences directly from CATH's Oracle database. A total of 86463 CATH domains were found. Afterwards, all CATH domains in the same CATH superfamily were grouped together in order to build a BLAST database of the sequences of three-dimensional structures specific to each of the CATH superfamilies.

### Generation of the groups of Gene3D sequences represented by the same CATH domain

In order to assign the closest CATH domain to each of the Gene3D sequences belonging to the same CATH superfamily, we queried each of the sequences in Gene3D against a database of CATH domains in that superfamily using BLAST. The best BLAST hit for each of the Gene3D sequences – provided the identity between the hit and the query was greater than 20% – was considered the closest CATH domain and hence the CATH domain was assigned as the structural representative of this sequence. After performing this classification for the whole set of sequences, all Gene3D sequences represented by the same CATH domain were grouped together and all the sequences within a group considered similar. A total of 2091 unique groups were generated.

### Alignment of the CATH domain groups using MUSCLE

During the previous step of the pipeline, the sequences contained in each of the groups of Gene3D sequences represented by the same CATH domain were considered similar. However, in order to collapse all the mutations from the Gene3D sequences onto the representative CATH domain sequence, the equivalence between pairs of residues needed to be established. To perform this task, multiple sequence alignments were constructed using the alignment package MUSCLE.

### Mapping SAAPdb mutations to CATH domain representative structures

The alignments generated by MUSCLE during the previous step of the pipeline were used to transfer the mutations, both pathogenic (PDs) and neutral (SNPs), from the sequences in Gene3D to the corresponding CATH structural representatives.

## Competing interests

The authors declare that they have no competing interests.

## Authors' contributions

Conceived the idea: AV, JMGI, CO, ACRM. Gathered the data and generated the mapping: JMGI, AB, LM, CY. All authors designed the server and its functionalities. Implemented the server: JMGI. Implemented the database: JMGI. Implemented the webservices: JMGI, AC. Wrote the paper: all authors. All authors read and approved the manuscript. CNIO covered the publication expenses.
